# Two-component dipolar Bose-Einstein condensate in concentrically coupled annular traps

**DOI:** 10.1038/srep08684

**Published:** 2015-03-03

**Authors:** Xiao-Fei Zhang, Wei Han, Lin Wen, Peng Zhang, Rui-Fang Dong, Hong Chang, Shou-Gang Zhang

**Affiliations:** 1 Key Laboratory of Time and Frequency Primary Standards, National Time Service Center, Chinese Academy of Sciences, Xi'an 710600, People's Republic of China; 2 Beijing National Laboratory for Condensed Matter Physics, Institute of Physics, Chinese Academy of Sciences, Beijing 100190, People's Republic of China; 3 College of Physics and Electronic Engineering, Chongqing Normal University, Chongqing 400047, People's Republic of China; 4 School of Electronics Engineering and Computer Science, Peking University, Beijing 100871, People's Republic of China

## Abstract

Dipolar Bosonic atoms confined in external potentials open up new avenues for quantum-state manipulation and will contribute to the design and exploration of novel functional materials. Here we investigate the ground-state and rotational properties of a rotating two-component dipolar Bose-Einstein condensate, which consists of both dipolar bosonic atoms with magnetic dipole moments aligned vertically to the condensate and one without dipole moments, confined in concentrically coupled annular traps. For the nonrotational case, it is found that the tunable dipolar interaction can be used to control the location of each component between the inner and outer rings, and to induce the desired ground-state phase. Under finite rotation, it is shown that there exists a critical value of rotational frequency for the nondipolar case, above which vortex state can form at the trap center, and the related vortex structures depend strongly on the rotational frequency. For the dipolar case, it is found that various ground-state phases and the related vortex structures, such as polygonal vortex clusters and vortex necklaces, can be obtained via a proper choice of the dipolar interaction and rotational frequency. Finally, we also study and discuss the formation process of such vortex structures.

Early studies of Bose-Einstein condensate (BEC) in dilute quantum gases demonstrate that contact interaction between atoms is the origin of most phenomena that have been observed in BEC^1^. Typically, this contact interaction is determined by the contact potential, which is characterized by the *s*-wave scattering length. However, a subsequent and recent achievement of dipolar BEC, especially for the realization of large magnetic moment(*µ* ≥ 6*µ_B_* with *µ_B_* being the Bohr magneton) atomic species, such as ^52^Cr (6*µ_B_*)^2^,^3^,^4^,^5^, ^168^Er (7*µ_B_*)^6^,^7^, and ^164^Dy (10*µ_B_*)^8^,^9^, paves the way towards a new fascinating research area, namely, that of degenerate dipolar gases.

The dipole-dipole interaction (DDI), is, contrary to the isotropic contact interaction present in condensates of alkali-metal atoms, long ranged, anisotropic, and it can be both attractive and repulsive. Therefore, it is predicted to induce novel ground-state properties and various interesting phenomena, making such system an ideal candidate for exploring a variety of nonlinear phenomena^10^,^11^,^12^,^13^,^14^,^15^. Meanwhile, attribute to the Feshbach resonance one can study the properties of a dipolar BEC for variable short-range contact interactions^16^,^17^,^18^,^19^. Recently, a spin-orbit-coupled dipolar BEC is also proposed by using Raman processes^20^, exhibiting novel features. Furthermore, progress towards the production of degenerate polar molecules with large electric dipole moments promises an exciting future in this research field^21^,^22^.

To date, there have been a number of theoretical studies on the ground state and elementary excitations of the dipolar gases in various external potentials, such as the harmonic trap^23^,^24^,^25^, optical lattice^26^,^27^,^28^, double-well^29^, toroidal trap^30^,^31^,^32^, ring-shaped trap and so on^33^,^34^,^35^,^36^. In these settings, the experimental and theoretical studies have shown that the static and dynamical properties of such systems are highly dependent upon the trap geometry. However, as far as we know, there has been little work on the dipolar gases in concentrically coupled annular traps.

To further explore the novel features caused by the DDI in this special trapping potential, in this report, we study the ground-state and rotational properties of a two-component dipolar condensate trapped in such a type of trap, taking into regard the contact interactions and DDI, together with the rotational frequency. This setting is modeled by the well-known coupled Gross-Pitaevskii (GP) equation with an additional nonlocal term accounting for the dipolar interaction. The ground-state structures and associated quantum phase transition, and rotational properties are investigated as a function of the ratio of dipolar to intra-component contact interactions and of the rotational frequency. We demonstrate that both the types of phase separation and the critical rotational frequency for the vortex formation are strongly influenced by the strength of the DDI. Due to the competition between the dipolar interaction and rotation, diverse exotic phases, such as polygonal vortex clusters and vortex necklaces are also observed.

## Results

### Model and the coupled Gross-Pitaevskii equations for the system

We consider a two-component dipolar BEC described by the macroscopic wave functions *ψ*_1_(*r*, *t*) and *ψ*_2_(*r*, *t*). The system contains atoms with magnetic dipole moments (labeled as component 1) and nonmagnetic atoms (labeled as component 2). In the mean-field framework, the ground state and dynamics of such a system can be well described by the following nonlocal coupled GP equations, 
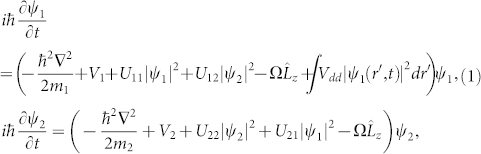
where *m_n_* and *V_n_* are the atomic mass and external potential for component *n* = 1 or 2, 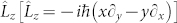 is the *z* component of the orbital angular momentum operator. The coefficients 
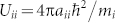
, and 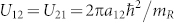 with *m_R_* = *m*_1_*m*_2_/(*m*_1_ + *m*_2_) being the reduced mass, denote the intra- and inter-component interaction, which are also related the *s*-wave scattering lengths *a_ii_* between atoms in the same component and *a*_12_ between atoms in different component, respectively. For simplicity, in this report we assume that the two components have the same mass *m*_1_ = *m*_2_ = *m* and the same number of atom *N*_1_ = *N*_2_. Furthermore, we consider *a_ij_* > 0, *i.e.*, repulsive short-range contact interactions, and set *U*_11_ = *U*_22_ = *U* (*a*_11_ = *a*_22_ = *a*). The external potential in the following shape is assumed to be equal to these two components, 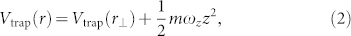
where *ω_z_* is the trapping frequency of the external potential along the *z* axis, and in the *x*-*y* plane the trap can be written as^37^,^38^, 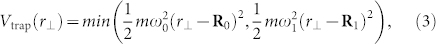
where two (overlapping) parabolas in *V*(*r*) with frequencies *ω*_0_ and *ω*_1_ are centered at the positions with *r*_⊥_ = **R**_0_ and *r*_⊥_ = **R**_1_, respectively, thus **R**_0_ and **R**_1_ indicate the two minima of such an external potential, and *ω*_0_ and *ω*_1_ are the harmonic trapping frequencies for these two different parabolas. The quasi-two-dimensional (2D) condensate is obtained by adding a very tight trapping potential along the *z*-axis (typically, *ω_z_*/*ω* = *λ* is chosen to be equal to 100 in this report and the wavefunction in the axial direction is in the Gaussian ground state), which completely freezes out the degrees of freedom of the gases along this direction, and hence 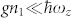 and 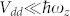. We note that the quantum-tunneling-related effects in vertically and concentrically coupled double-ring traps were studied in^37^,^39^,^40^,^41^, and the concentric ring structures were extensively studied in topics of electronics in quantum rings and in semiconductor heterostructures^42^.

In order to ensure that the outer ring is more tight and the product of the “width” of each annulus times the radius of each annulus to be comparable to each other, we set *ω*_1_ > *ω*_0_. It is noteworthy that the system will show similar behavior for different values choices of **R**_0_, **R**_1_, *ω*_0_, and *ω*_1_, as long as *ω*_1_ > *ω*_0_. Thus, without loss of generality, we special to the case with **R**_0_ = 2*a*_0_ and **R**_1_ = 4*a*_0_ with 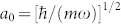 being the oscillator length, *ω* = *ω*_0_/4, and *ω*_1_/*ω*_0_ = 5/4. Finally, after integrating over the profile along *z* axis, the corresponding effectively 2D contact-like interaction becomes 
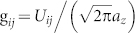
 with 
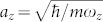
 being the oscillator length in the *z* direction, which can be attributed to the compression along the *z* axis.

The last term of the first equation of Eq. (1) describes the effect of the nonlocal DDI, and has the form 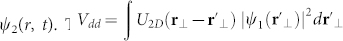 with 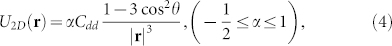
where for magnetic dipoles *C*_dd_ = *µ*_0_*µ*^2^/(4*π*) (for electric dipoles we have *C*_dd_ = *d*^2^/4*πε*_0_ with *d* being the electric dipole moment) with *µ*_0_ and *µ* being the magnetic permeability of vacuum and the magnetic dipole moment of the atom, respectively. *θ* is the angle between the polarization axis and the vector **r** between the positions of the two dipoles (that is, cos *θ* = **n** · **r**/|**r**|). As an initial effort to understand the rich physics of such a system, in this report we try to simplify the situation by considering a special case where the effective dipoles are polarized along the rotation axis, which is also the symmetrical axis of the trap^43^,^44^.

**Figure 1:**
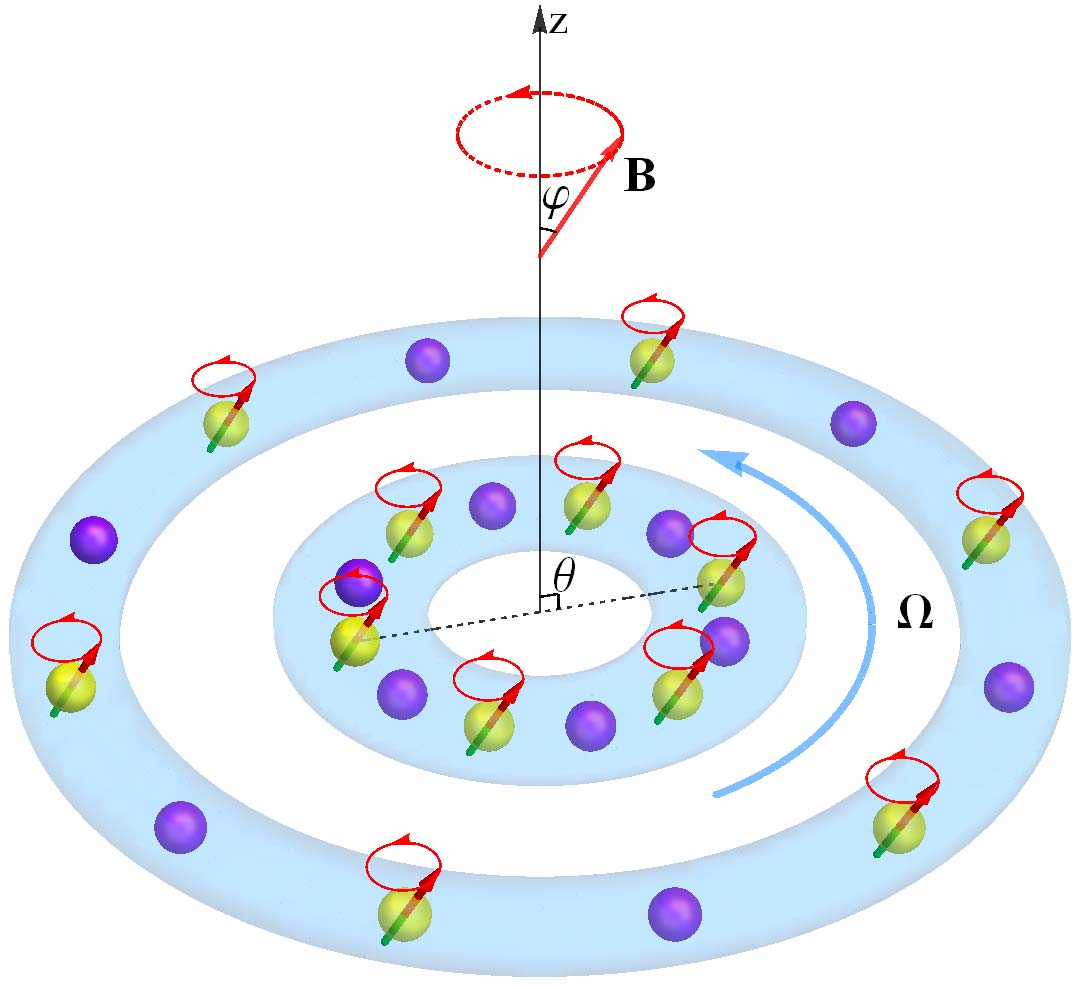
Schematic illustration of the physical system under consideration. Here component 1 (yellow) has a dipole moment, while component 2 (purple) hasn't. The dipole moment is polarized by a rotating external field B, forming an angle *ϕ* with the *z* axis. The angle between the effective polarization axis and the vector between the positions of the two dipoles is set as *θ* = *π*/2. All the atoms move on the *x*-*y* plane with an angular frequency Ω, which is along the *z* axis.

It is noteworthy that the tunable parameter, *α* = (3 cos^2^ *ϕ* − 1)/2, can be changed continuously from −1/2 to 1, by means of rotating orienting field. In this case, the dipoles are rapidly rotated around the polarized axis *z*, forming a tunable angle *ϕ*. Here we note that the rotation frequency must be much smaller compared with the Larmor frequency *ω*_Larmor_, but much larger than the trapping frequencies; hence, the dipoles can adiabatically follow^45^. This tunability providing the possibility to change the dipolar interaction from repulsive to attractive, and was used to investigate the two-dimensional bright soliton in dipolar BECs^43^. It also becomes crucial in the control of the types of phase separation of two-component dipolar BEC in concentrically coupled annular traps, as discussed below. Figure 1 shows schematically the physical system under consideration.

For dipolar condensate with also contact interactions, it is useful to introduce a dimensionless parameter to characterize the relative strength of the dipolar and *s*-wave interaction, 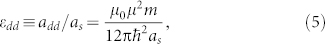
where *a_s_* is the *s*-wave scattering length for contact interaction of component 1, hence the “dipole length” *a_dd_* can be defined as 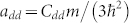, then *ε_dd_* = 4*πC_dd_*/3*U*_0_. Since 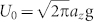, *ε_dd_* is also equal to 
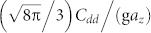
. Here we want to note that for *ε_dd_* < 1, the short-range part of the interparticle interaction dominates and DDI provides only corrections. This case corresponds to a stable BEC and was studies in earlier experiments with ^52^Cr BEC (*ε_dd_* ≈ 0.16), in which the correction due to magnetic DDI between ^52^Cr atoms was measured to be of the order of 0.1. Also, this value has been further increased to 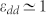 by using a Feshbach resonance to reduce *a_s_*^4^. Very recently, there has been the exciting achievement of the condensation of hereto-nuclear molecules in their ground rovibrational state, which can have large electric dipole moments, leading to very strong electric dipole interactions^46^.

### Ground state and phase transition for nonrotational case

As is well known, by varying the contact interactions, a two-component BEC containing only repulsive contact interactions and loaded in a concentric double annular trap shows a series of ground-state phases, such as azimuthal and radial phase separation, together with the familiar phase coexistence. In what follows we first perform a series of numerical experiments to study the effects of dipolar interaction on the ground-state structures and the associated phase transition for the nonrotational condensate. Without loss of generality, we select three typical sets of parameters for the contact interactions: (i) g = 5, g_12_ = 55, (ii) g = 15, g_12_ = 55 and (iii) g = 60, g_12_ = 55 to reveal the effects of modifying the strength of the dipolar interaction^37^,^38^.

**Figure 2:**
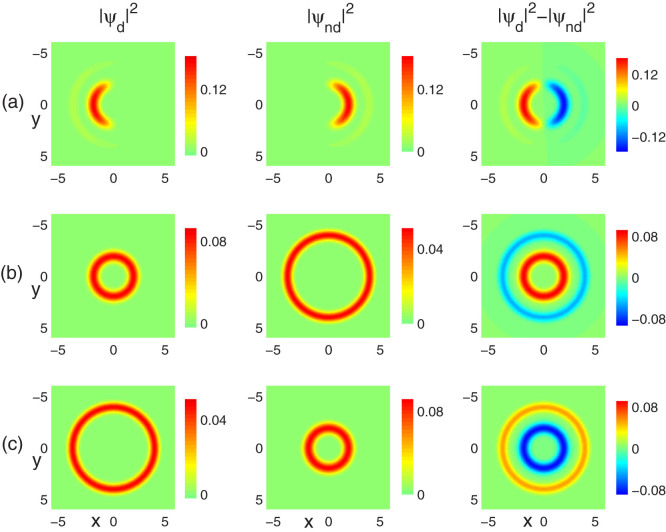
The types of phase separation as a function of the dipolar interaction. By modifying the strength of the dipolar interaction, the types of phase separation undergo transitions from azimuthal (a) to inner-outer radial (b), and then to outer-inner radial (c) separation. Here the contact interactions are fixed at g = 5, g_12_ = 55, and the aspect ratio of the potential is *λ* = 100. The strengths of dipolar interaction are set as (a) *ε_dd_* = 0, (b) *ε_dd_* = −0.3 and (c) *ε_dd_* = 0.2, respectively. The field of view in each panel is 6.4 × 6.4 in units of *a*_0_.

Figure 2 shows the typical ground-state density profiles of a non-rotating two-component dipolar condensate for fixed contact interactions g = 5 and g_12_ = 55, but for varied dipolar interactions. In the absence of DDI, the small value of the intra-component interaction cannot compensate for the stronger confinement of the outer ring; hence the two components occupy mainly the inner ring and the system is close to being quasi-one-dimensional and shows azimuthal phase separation, as shown in Fig. 2(a). On decreasing *ε_dd_* to −0.3, we find a phase transition between azimuthal phase separation and radial phase separation. That is, the component 1 still stays in the inner ring due to the net attractive dipolar interaction, while component 2 is pushed out toward the outer ring due to the effectively repulsive interaction between these two components. Typical density profiles for the two components are shown in Fig. 2(b). However, if *ε_dd_* is positive, the situation is the opposite. As shown in Fig. 2(c) for *ε_dd_* = 0.2, we find that component 1 occupies mainly the outer ring, while the inner ring for the other component. Actually, the explanation of the above phenomenon lies in the well-known fact that the interactions are known to be the predominant factors which can affect the ground-state density profile of a two-component condensate. The DDI, which is purely repulsive in this situation, drastically increases or decreases the effectively contact-like interactions. Here we want to note that the result still holds for larger value of *ε_dd_*.

**Figure 3:**
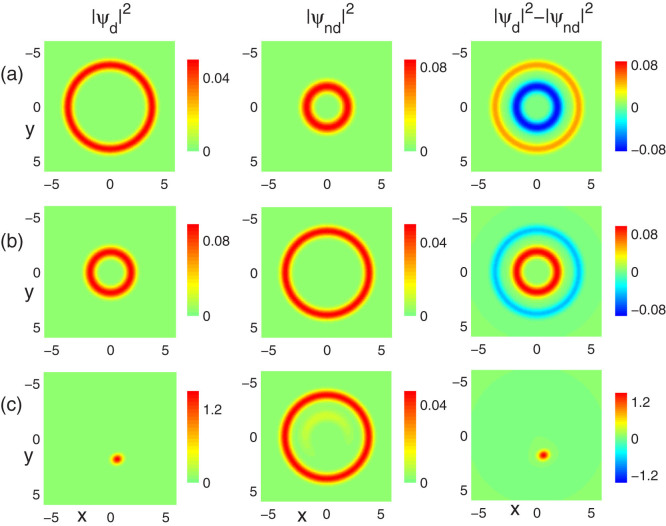
Symmetry breaking induced by the dipolar interaction. The ground-state density profiles of a non-rotating two-component dipolar BEC for fixed contact interactions g = 15, g_12_ = 55, but for different dipolar interactions (a) *ε_dd_* = 0, (b) *ε_dd_* = −1.23 and (c) *ε_dd_* = −1.46, respectively. The aspect ratio of the potential is *λ* = 100. The field of view in each panel is 6.4 × 6.4 in units of *a*_0_.

Increasing the intra-component interaction g to 15, the coupled system is initially in radial phase separation, as shown in Fig. 3(a). Interestingly, in this case if we decreases *ε_dd_* to −1.23, we find an exchange of the location of each component, as shown in Fig. 3(b). On further decreasing the value of DDI, such as *ε_dd_* = −1.46, the dipolar component is further compressed into a small droplet due to the strongly dipolar interaction. As a result, the rotational symmetry is broken, as shown in Fig. 3(c). For the positive value of DDI, we cannot observe a similar structural change even for larger positive *ε_dd_* compared with the former case. This can be understood by the fact that the outer ring potential can always trap component 1 although its repulsive interaction is increasing.

**Figure 4:**
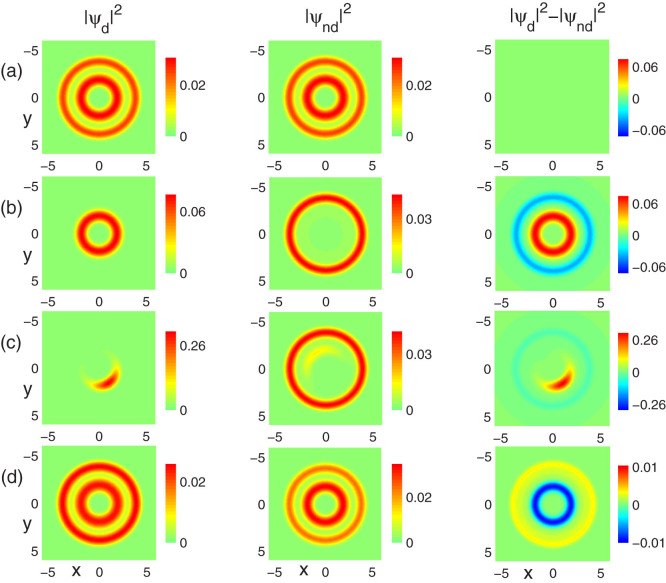
Miscibility-immiscibility transition induced by dipolar interaction. The ground-state density profiles of a non-rotating two-component dipolar BEC for fixed contact interactions g = 60, g_12_ = 55, but for different dipolar interactions (a) *ε_dd_* = 0, (b) *ε_dd_* = −0.34, (c) *ε_dd_* = −0.87 and (d) *ε_dd_* = 0.8, respectively. The aspect ratio of the potential is *λ* = 100. The field of view in each panel is 6.4 × 6.4 in units of *a*_0_.

Figure 4 shows the situation for fixed contact interactions g = 60, g_12_ = 55, but for varied dipolar interactions. In this case, the system is initially in phase coexistence. A decrease of the DDI leads to the accumulation of dipolar atoms in the inner ring, which is similar with the radial phase separation case. Typical examples are shown in Figs. 4(b) and 4(c) for *ε_dd_* = −0.34, −0.87, respectively. However, if we change *ε_dd_* from negative to positive, the system still stays in phase coexistence with no phase transition occuring, as shown in Fig. 4(d) for *ε_dd_* = 0.8. Due to the repulsive nature of the DDI, part of the nondipolar atoms are repelled to the outer ring, as compared with Fig. 4(a).

Given the results given above, we thus conclude that the tunable dipolar interaction can be used to control the types of phase separation, and can also lead to spontaneous rotational symmetry breaking. This behavior is in a sense reminiscent of the symmetry breaking and self-trapping of a dipolar BEC confined in a double-well potential^29^, if the inner or outer rings is regarded as the left or right well of the double-well potential. Finally, we have checked other values of DDI, and the results are qualitatively similar to the above phenomenon. Thus, the results presented above are representative of the possible ground state phases and the related phase transitions.

### Rotational properties

Another important issue that has always attracted much interest in the field of quantum gases is the superfluid character of the condensate. The presence of quantized vortices is a clear signature of superfluidity^30^. At first sight, the rotational properties of a multi-component gas may look like a trivial generalization of the case of a single component. However, as long as the different components interact and exchange angular momentum, the extra degrees of freedom associated with the motion of each component is not at all a trivial effect. It is found that vortex lattices in single rotating atomic BEC with dipole interaction can display the triangular, square, stripe, and bubble phases^47^,^48^. In a two-component system, the vortex states of square, triangular, double core and serpentine lattices are showed according to the intercomponent coupling constant and the geometry of trap^49^,^50^. In what follows we first consider the nondipolar case with varied rotational frequency for fixed contact interactions, and then move to the dipolar one.

### Vortex structure for nondipolar condensate

**Figure 5:**
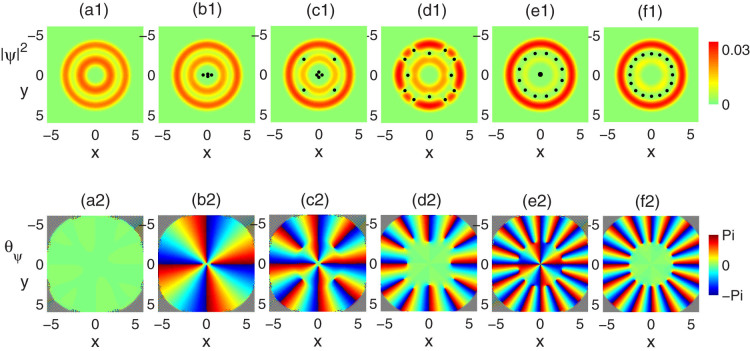
Typical vortex structures of a two-component nondipolar BEC. Here the upper plots [(a1)-(f1)] show the densities of either one component of the system (the marked dots denote the locations of the vortices), and the lower plots [(a2)-(f2)] indicate the corresponding phases. The aspect ratio of the potential *λ* = 100, and the contact interactions are fixed as g = 100, g_12_ = 150. The rotational frequencies are set as (a) Ω = 0.54, (b) Ω = 0.55, (c) Ω = 0.68, (d) Ω = 0.7, (e) Ω = 0.8 and (f) Ω = 0.9, respectively. Note that, without dipolar interaction, these two components act like one component condensate, hence we only plot one component, either 1 or 2. The field of view is 6.4 × 6.4 in units of *a*_0_.

Figure 5 shows the typical ground-state density and phase distributions of a two-component nondipolar condensate for fixed contact interactions, but for varied rotational frequencies. Since g_11_ = g_22_ = g is assumed, the two-component Bose gas behaves like a one-component one. As a result, only one component is shown in this figure. In our simulations, we find that there exists a critical value of Ω*_c_*, above which vortex state can first forms at the trap center. For the parameters used here, we find Ω*_c_* ≈ 0.55, as shown in Fig. 5(b) [Fig. 5(a) is plotted for comparison]. From this figure, we can observe that when the rotational frequency approach the critical value, four vortices appear at the trap center, forming a necklace structure. Moreover, we also have examined other contact interaction parameters, and find that the value of the critical rotational frequency decreases with the increasing of the strength of contact interactions. Finally, for the vortices located at the center of the trapping potential, we call them “hidden” vortices, which were previously studied in a rotating double-well potential^51^,^52^. Due to the central barrier, the amplitude of the density of the wavefunction at this region is almost negligible, thus these hidden vortices can not be directly seen from the density distribution. We note that with a little increase of the rotational frequency, no changes made to such vortex structure.

If we increase the rotational frequency Ω to 0.68, vortices begin accumulate at the low-density region between the inner and outer rings, and also form a necklace structure. As is well -known, it is energetically favorable for the vortices to site in the low-density regions. Hence we first observe the formation of vortices at such region. Typical density and phase distributions of such case is shown in Fig. 5(c). By further increasing the strength of the rotational frequency, as shown in Fig. 5(d) for Ω = 0.7, it is interesting to find that eight visible vortices are formed at the outer ring and four vortices are located at the region between two rings. It is necessary to point out the following: (i) the previous four hidden vortices located at the center of the trapping potential gradually penetrate the central barrier and arrange themselves in a ring between the inner and outer rings; (ii) the previous four vortices located between the inner and outer rings also penetrate the potential and eventually arrange themselves in a ring with the other four new nucleated vortices at the outer ring. These phenomena can be understood by the fact that the increases of rotational frequency enhances the centrifugal force, and more and more atoms are repelled to the outer ring. Its also be verified by our real-time propagation of the wavefunction.

For even higher rotational frequencies, such as Ω = 0.8, 0.9 shown in Figs. 5(e) and 5(f), more and more visible vortices are nucleated at the region between both rings, and form a necklace structure. More specifically, there are 12 and 16 visible vortices for Figs. 5(e) and 5(f), respectively. Moreover, there is a high-order hidden vortices located at the trap center in Fig. 5(e). From the above analysis, it is not difficult to speculate that such vortices will gradually penetrate the barriers and evolve into the visible one with increases of the rotational frequency [see Fig. 5(f)].

### Vortex structure for dipolar condensate

Next we consider the effects of DDI on the rotational properties of the dipolar condensate. In this case, due to the presence of DDI, the two components no longer show the same behavior. To highlight the effects of DDI and rotation, the vortex structures are explored through tuning the strength of DDI and the rotational frequency of the system. Our numerical results show that the density distributions and vortex structure of the system are strongly dependent on the strengths of both DDI and the rotating frequency.

**Figure 6:**
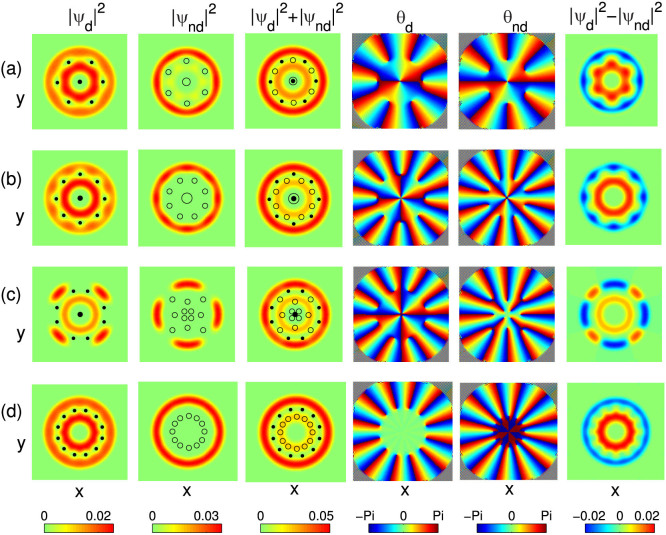
Typical vortex structures of a two-component dipolar BEC. The aspect ratio of the potential *λ* = 100, and the contact interactions are fixed as g = 100, g_12_ = 150. The rotational frequencies and dipolar interactions are set as (a) Ω = 0.54, *ε_dd_* = 1.2, (b) Ω = 0.66, *ε_dd_* = 0.9, (c) Ω = 0.7, *ε_dd_* = 0.3 and (d) Ω = 0.7, *ε_dd_* = 0.8, respectively. In the density distribution, the marked dots and circles denote the locations of the vortices for dipolar and nondipolar components, respectively. Note that the fourth and fifth columns are the phases of dipolar and nondipolar condensates, respectively, and the sixth one is the density difference of such two condensates. The field of view is 6.4 × 6.4 in units of *a*_0_.

Interestingly, we observe that vortices and its related vortex clusters can be induced by the repulsive DDI even the rotational frequency is below the critical one for the nondipolar case [see Fig. 6(a) for Ω = 0.54 and *ε_dd_* = 1.2]. Meanwhile, the ground-state phase of the system changes from phase coexistence to radial phase separation, as shown in the last column of Fig. 6(a) for the density difference of these two components. Furthermore, we observe the formation of interlaced honeycomb vortex structure for each component, which can also be understood by the similar argument discussed for the nondipolar case: increasing of the strength of DDI leads to the increases of the repulsive intra-component interaction, but to the decreases of the critical rotational frequency.

Fig. 6(b) shows the similar phase transition and the octagonal vortex cluster structure for such a system, but for a higher rotational frequency Ω = 0.66 with *ε_dd_* = 0.9. Compared with the former case, we observe same phase transition (from phase coexistence to radial phase separation), but different vortex cluster (interlaced octagonal vortex cluster in present case), which can be attributed to the higher rotational frequency. Here we want to emphasize that in these two cases, the nucleated vortices form a regular polygonal structure, no matter interlaced honeycomb or octagonal vortex clusters. In addition, we also have examined the vortex structures for other DDI parameters in these two cases, and found the similar vortex structure.

Figs. 6(c)–6(d) plot the density and phase distributions for a more higher fixed rotational frequency Ω = 0.7. It is found that for a fixed rotational frequency, the system exhibits different ground-state phases for different strengths of DDI. Typical examples are shown in Fig. 6(c) for *ε_dd_* = 0.3 and Fig. 6(d) for *ε_dd_* = 0.8, where the system is in azimuthal and radial phase separation, respectively. The different ground-state phases are also reflected in the density difference of such two components, which is shown in the last column of each plot. Moreover, the nucleated vortices form other interesting vortex structures, such as interlaced vortex necklace. Furthermore, by comparing Figs. 6(c) and 6(d), we further find the number of vortices increases with the strength of DDI.

Given the above analysis, we conclude that the DDI can be used not only to control the phase separation, but also to induce various polygonal vortex clusters and vortex necklaces. Finally, we note that it becomes increasingly difficult to find the lowest-energy stable structure when we further increase the rotational frequency. We plan to study this problem in greater detail in a future work.

## Discussion

We now show that ^52^Cr (^164^Dy or ^168^Er) is a candidate for observing the described effects in experiment. In this case, dipolar component 1 and nondipolar component 2 consist of states with spin projections *m_J_* = −*J* and *m_J_* = 0, respectively. The typical particle number is about 10^3^–10^5^, and the unit length 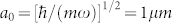, which is the typical unit of length in BEC experiments. Thus, the typical radii of these two rings are **R**_0_ = 2 and **R**_1_ = 4 *µm*, respectively, the typical distance between the two rings is 2 *µm*, and the typical atom density is about 1.2 × 10^12^. The next step is to tune the two-body interactions between atoms, including the usual contact interactions and the DDI. For the nondipolar case, in realistic physical systems, the interactions between atoms can be controlled by modifying atomic collisions, which are experimentally feasible due to the flexible and precise control of the scattering lengths achievable by *magnetically* tuning the Feshbach resonances. For the dipolar component 1, tuning the dipolar interaction must be combined with a reduction of the contact interaction via the *optical* Feshbach resonances^43^,^53^ [see ref. 53 for detailed parameter values]. Within current experimental techniques, the static, *in situ* sizes for a trapped condensate can be experimentally realized. However, as far as we know, it may be difficult to observe the locations of vortices *in situ* due to its small sizes, but it would become easier to observe in time-of-flight free expansion. Most recently, the signs of solitonic vortices were observed by Donadello *et al.* by using a method of twisted densities^54^. Moreover, the vortex gyroscope imaging method was used by Powis to simultaneously detect the locations and signs of multiple quantized vortices in a BEC^55^.

In summary, within the frame of mean-field theory, we have investigated the ground-state and rotational properties of a rotating two-component dipolar BEC confined in concentrically coupled annular traps. We identify the states where the two components coexist, or separate, either radially or azimuthally, as a function of the ratio of dipolar to intra-component contact interactions, and of the rotational frequency. Our results show that the tunable dipolar interaction can be used to control the location of each component, and to induce desirable phase transition among these three different ground-state phases. We also discuss the vortex structure of such a two-component system for nondipolar and dipolar cases, and find various interesting vortex structures, such as interlaced honeycomb and octagonal vortex clusters, as well as vortex necklaces. These results show that the DDI has considerable effects on the ground state and vortex structures of condensate of the alkali metal atoms even in concentrically coupled annular traps. Such tunable dipolar interaction provides a powerful tool for exploring the rich physics of dipolar degenerate quantum gases.

A natural extension of this report is to try to generalize the ideas presented herein to the case where the dipoles are aligned along some arbitrary and tunable direction, and to the spinor condensate. More specifically, in the former case, the anisotropic nature of the dipolar interaction can cause novel properties, and more complicated pattern can be formed. For the latter case, the interplay between the dipolar and the spin-exchange interactions can induces a rich variety of quantum phases that exhibit spontaneous magnetic ordering in the form of intricate spin textures.

## Methods

Contrary to the usually employed GP equation with short-range contact interactions, the evaluation of the integral term in the first equation of Eq. (1) deserves special attention^56^,^57^,^58^. The integral over the dipolar potential is evaluated in Fourier (momentum) space by a convolution identity requiring the Fourier transformation of the dipolar potential and the condensate density. More specifically, the Fourier transformation of the dipolar potential is evaluated analytically, and the remaining Fourier transformation are evaluated numerically by using a fast Fourier transformation algorithm. Employing the convolution theorem, the Fourier transform of the dipole potential, 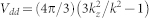, and integrating over the *z* direction, we arrive at the effective nonlocal GP equation for dipolar component 1: 
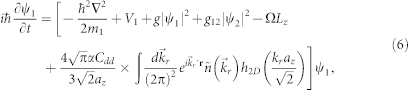
where 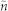 is the Fourier transform of *n*(**r**) = |*ψ*_1_(**r**)|^2^, and 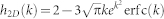, with erfc(*x*) the complementary error function. The energy per density functional in the rotating frame has the standard GP form but with a new term, *E*_dip_, which is the interaction energy due to the magnetic DDI, 
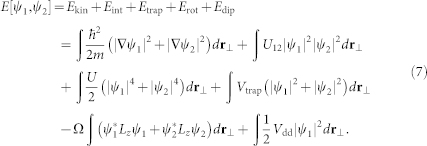
The ground-state solutions can be obtained from the minimization of the total energy (7). In our numerical calculations, we adopt the split-step method within an imaginary-time propagation (*t* → *it*) approach. The minimization procedure continues until the fluctuation in the norm of the wave function becomes smaller than 10^−7^. We also have checked our numerical results with different initial wave functions, including the ground state of the system trapped in a stationary harmonic potential and a randomly generated one. The real ground-state solutions are obtained by comparing the computed energies. Finally, it is convenient to introduce the scales characterizing the trapping potential: the length, time, and wave function are scaled as 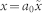, 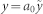, 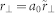, 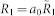, 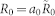, 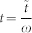 and 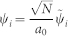, respectively, with 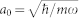.

## Author Contributions

S.G.Z. conceived the idea and supervised the overall research. X.F.Z., W.H., L.W. and P.Z. designed and performed the numerical experiments. R.F.D. and H.C. analyzed numerical results. X.F.Z. wrote the paper with helps from all other co-authors.
